# Enhancing prognostic accuracy in sepsis: a modified SOFA score incorporating lymphocyte count as an immune function marker

**DOI:** 10.3389/fcimb.2025.1593589

**Published:** 2025-07-31

**Authors:** Zhishan Huang, Ying Cui, Chen Zhang, Huijie Yu, Lina Chen, Xingxing Wang, Jiancang Zhou, Peng Lan

**Affiliations:** ^1^ Department of Critical Care Medicine, Sir Run Run Shaw Hospital, Zhejiang University School of Medicine, Hangzhou, China; ^2^ Department of Critical Care Medicine, The Fourth Affiliated Hospital, Zhejiang University School of Medicine, Yiwu, China; ^3^ Department of Emergency Medicine, The First Hospital of Jiaxing, Jiaxing University, Jiaxing, China

**Keywords:** sepsis, SOFA score, lymphocyte count, mortality, immune

## Abstract

**Background:**

Sepsis, characterized by organ dysfunction due to a dysregulated immune response, is diagnosed using the Sequential Organ Failure Assessment (SOFA) score, which currently lacks immune function markers. The objective of this research is to enhance the predictive precision of SOFA for sepsis by integrating lymphocyte count as an indicator of immune system functionality.

**Methods:**

This retrospective study was based on the MIMIC-IV database. The absolute lymphocyte count (ALC) was assessed as a predictive biomarker through multivariate analysis utilizing the Cox proportional-hazards model and was integrated into a modified SOFA score (ALC-SOFA). Associations between ALC-SOFA score and mortality were assessed using Kaplan-Meier survival analysis. Predictive performance was evaluated by comparing the area under the receiver operating characteristic (AUROC) curve for ALC-SOFA and the original SOFA score. The primary endpoint was the mortality rate at 28 days, with additional secondary endpoints including the mortality rates at 7 and 90 days.

**Results:**

10,709 patients with sepsis were included in this study. ALC was significantly lower in nonsurvivors than in survivors (0.90 ± 0.62×10^9^/L vs. 1.12 ± 0.69×10^9^/L, P<0.001). Patients with lower absolute lymphocyte counts (ALC) exhibited a significantly higher risk of 28-day mortality (HR = 0.62, P<0.001). Survival analysis revealed higher mortality rates with increasing ALC-SOFA scores. The ALC-SOFA score demonstrated improved prognostic performance for 28-day mortality (AUROC = 0.680 vs. 0.664, P<0.001) and 90-day mortality (AUROC = 0.666 vs. 0.647, P<0.001) compared to the original SOFA score.

**Conclusion:**

Incorporating ALC into the SOFA score significantly improves its ability to predict sepsis-related outcomes. The ALC-SOFA score provides a novel tool for prognostic assessment, highlighting the critical role of immune function in sepsis management.

## 1 Introduction

Sepsis is characterized by a life-threatening dysfunction of organs resulting from a dysregulated immune response to the infection (Singer et al., 2016). Worldwide, sepsis accounted for roughly 48.9 million incidents and led to approximately 11.0 million fatalities in 2017, underscoring its significant impact on global healthcare systems (Rudd et al., 2020).

The 2016 International Consensus Definition for Sepsis and Septic Shock (Sepsis-3) characterizes sepsis as the presence of organ dysfunction, manifested by an acute elevation of at least 2 points in the Sequential Organ Failure Assessment (SOFA) score, attributable to the infectious process (Singer et al., 2016). The SOFA score was developed in 1996 and has since evolved into one of the most frequently employed scoring methods in intensive care units, with clinical applications including the assessment of organ dysfunction severity, monitoring of disease progression. It is also widely used in research for risk stratification and evaluation of treatment outcomes in critically ill patients (Vincent et al., 1996; Cour et al., 2016; Raith et al., 2017). However, despite its utility, the SOFA score does not include markers of immune function, which belie the complex and multifaceted immune responses involved in sepsis (Wiersinga and van der Poll, 2022). Although various modified SOFA scores have been proposed in previous studies (Lee et al., 2022; Park et al., 2023; Wang et al., 2023), they have not integrated immune biomarkers into prognostic models. Recent studies emphasize the value of immune biomarkers in sepsis prognosis. Fang et al. proposed an immune dysfunction score combining cytokines, HLA-DR, and neutrophil-to-monocyte ratios, improving 28-day mortality prediction versus SOFA (AUROC 0.853 vs. 0.789) (Fang et al., 2017). Zhou et al. showed that IL-10 enhanced early sepsis prediction when added to NEWS, and IL-6 independently predicted 28-day mortality (Zhou et al., 2024). These findings show that immune markers reveal important immune changes in sepsis that are linked to patient outcomes but are not captured by organ-based scores like SOFA. This indicates the need to update the SOFA score by incorporating markers of immune function.

Absolute lymphocyte count (ALC) is a simple and clinically accessible indicator that reflects immune profiles and predicts outcomes in sepsis patients (Yang et al., 2024). The continuation of lymphopenia following the diagnosis of sepsis serves as a forecaster of both early and late mortality, and it may function as a biological indicator of the immunosuppressive state induced by sepsis (Drewry et al., 2014). Given the limitations of the SOFA score and the simplicity of ALC, we propose integrating ALC into the SOFA score. This study aims to develop a modified SOFA scoring system that incorporates ALC as a marker of immune function, to improve its predictive accuracy for sepsis-related outcomes.

## 2 Materials and methods

### 2.1 Data source

This study used the data from the MIMIC-IV (v3.1) database, an extensive, openly accessible resource developed by the Laboratory of Computational Physiology at the Massachusetts Institute of Technology (Johnson et al., 2023). The database is publicly available at https://mimic.mit.edu/. The database comprises information on all patients who were hospitalized at the Beth Israel Deaconess Medical Center from 2008 to 2022. It includes a wide range of data such as laboratory test outcomes, medication regimens, vital signs, the duration of hospital stays, and other clinical details. Authorized access to the database was obtained by one of the authors, who was responsible for extracting the data (Record ID: 66433833). To ensure patient privacy, all personal identifiers were removed and replaced with randomized codes. Consequently, this research did not require ethical approval and informed consent.

### 2.2 Patient population and data extraction

**Figure 1:**
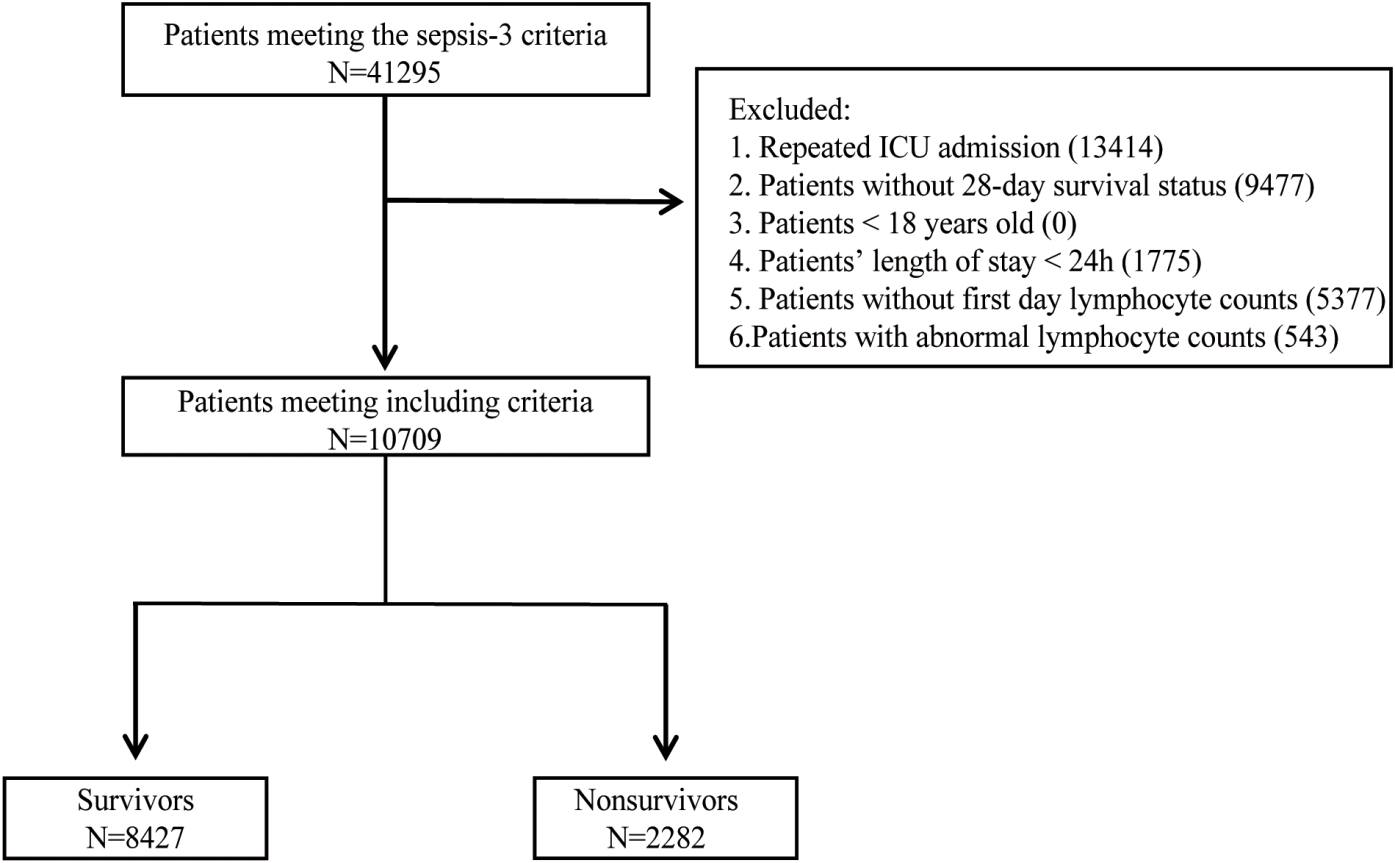
Flowchart of the enrolled patients.

The flowchart outlining the procedure for selecting the study population was present in **Figure 1**. The diagnosis of sepsis was established by the Third International Consensus Definition for Sepsis (Sepsis-3) (Singer et al., 2016). Septic shock was diagnosed in patients with sepsis who required vasopressor treatment during their hospitalization. A total of 41,295 patients met the Sepsis-3 criteria. After excluding those with repeat hospital admissions, missing 28-day survival status, under 18 years of age, the duration of ICU stay ≤ 24 hours, missing lymphocyte counts on the first day, and abnormal lymphocyte count values (according to Tukey’s fences), a total of 10,709 patients were eligible for the study. Patients were categorized into two groups: survivors (N=8427), comprising those who survived 28 days of ICU admission, and nonsurvivors (N=2282), comprising those who died within 28 days of ICU admission. Extracted variables included sex, age, race, administration of vasopressors during hospitalization, first-day SOFA score, first-day laboratory data, comorbidities, first-day vital signs, and infection sites. The vasopressors included dopamine, epinephrine, norepinephrine, phenylephrine, vasopressin, dobutamine, and milrinone. The initial laboratory results on the first day encompassed measurements of hemoglobin, platelets, albumin, bilirubin, blood urea nitrogen (BUN), creatinine, alanine aminotransferase (ALT), aspartate aminotransferase (AST), international normalized ratio (INR), activated partial thromboplastin time (APTT), white blood cell (WBC) count and ALC. Except for ALC taking the minimum value, the rest of the laboratory data took the maximum value. Comorbidities, identified by ICD-9 and ICD-10 codes, included diabetes mellitus, hypertension, acute kidney failure, chronic kidney disease, chronic pulmonary disease, chronic liver disease, and cardiomyopathy. The initial vital signs recorded on the first day comprised systolic blood pressure (SBP), diastolic blood pressure (DBP), mean arterial pressure (MAP), heart rate, respiratory rate, and body temperature, with mean values taken for each. Based on positive microbiological culture results, the sites of infection identified included the bloodstream, respiratory tract, urinary system, gastrointestinal tract, peritoneum, pleura, skin and soft tissues. The status of HIV infection, chemotherapy, and immunosuppressant use was determined based on ICD-9 and ICD-10 codes. Structured Query Language (SQL) with PostgreSQL 15.4 was used to extract data from MIMIC-IV.

### 2.3 Statistical analyses

The primary endpoint was 28-day survival and secondary endpoints were 7-day and 90-day survival. Continuous variables that are normally distributed are presented as the mean ± standard deviation, whereas that do not follow a normal distribution are presented as the median accompanied by the interquartile range (IQR). Categorical variables were summarized as the number (%). For comparisons, continuous variables were analyzed using one-way ANOVA for normally distributed data and the Kruskal-Wallis test for non-normally distributed data, while categorical variables were analyzed using chi-square tests. The Cox proportional-hazards model was utilized for both univariate and multivariate analyses to evaluate the impact of various factors on the primary endpoint (Cox, 1972). The predictive efficacy of the initial and revised SOFA scores was evaluated by contrasting the area under the receiver operating characteristic (AUROC) curve and hypothesis testing was conducted using the DeLong test. An improvement in risk prediction for revised SOFA scores is assessed by the continuous net reclassification improvement (NRI) and integrated discrimination improvement (IDI) indices. 95% confidence intervals (CIs) were calculated using bootstrapping with 1000 replicates. Survival was estimated by the Kaplan-Meier method, and any differences in survival were evaluated with the log-rank test. The P value of 0.05 or less was considered statistically significant for determining differences. All statistical tests were two-tailed, and analyses were conducted using R version 4.4.1 (R Foundation for Statistical Computing, Vienna, Austria).

## 3 Results

### 3.1 Basic characteristics

In this study, a total of 10,709 patients diagnosed with sepsis were enrolled, with 8,427 (78.7%) surviving beyond 28 days and 2,282 (21.3%) succumbing within 28 days after ICU admission (**Table 1**). Males accounted for 57.7% of the total cohort, with a higher proportion among survivors compared to nonsurvivors (P = 0.020). The median age of the total cohort was 67.50 years, with nonsurvivors being significantly older than survivors (72.59 years vs. 66.58 years, P < 0.001). Nonsurvivors had higher first-day SOFA scores compared to survivors (8.0 vs. 5.0, P < 0.001) and had a higher proportion of vasopressor use than survivors (59.8% vs. 47.2%, P < 0.001).

**Table 1:** Baseline characteristics of the patients with sepsis.

Characteristics	Total (N=10709)	Survivors (N=8427)	Nonsurvivors (N=2282)	*P*-value
Male, No. (%)	6182 (57.7)	4914 (58.3)	1268 (55.6)	0.020
Age (IQR), years	67.50 (56.37-78.90)	66.58 (55.13-77.54)	72.59 (61.03-82.92)	<0.001
Race, No. (%)				<0.001
White	6897 (64.4)	5535 (65.7)	1362 (59.7)	
Asian	323 (3.0)	266 (3.2)	57 (2.5)	
Black	890 (8.3)	734 (8.7)	156 (6.8)	
Hispanic/Latino	317 (3.0)	265 (3.1)	52 (2.3)	
Others	775 (7.2)	589 (7.0)	186 (8.2)	
Unknown	1507 (14.1)	1038 (12.3)	469 (20.6)	
Vasopressor use, No. (%)	5345 (49.9)	3981 (47.2)	1364 (59.8)	<0.001
SOFA Score (IQR)	6.0 (4.0-8.0)	5.0 (3.0-8.0)	8.0 (5.0-11.0)	<0.001
Laboratory Data (mean ± SD)				
Hemoglobin, g/dl	11.51 (2.21)	11.57 (2.16)	11.28 (2.37)	<0.001
Platelets, 10^3^/L	222.01 (121.58)	221.65 (117.24)	223.32 (136.46)	0.562
Albumin, g/dL	3.19 (0.53)	3.22 (0.50)	3.08 (0.61)	<0.001
Bilirubin, mg/dL	2.20 (3.87)	1.96 (2.99)	3.08 (6.03)	<0.001
BUN, mg/dL	33.35 (26.49)	30.72 (24.75)	43.06 (30.19)	<0.001
Creatinine, mg/dL	1.83 (1.85)	1.74 (1.87)	2.13 (1.73)	<0.001
INR	1.72 (1.25)	1.62 (1.12)	2.06 (1.60)	<0.001
APTT, second	45.03 (30.14)	43.25 (28.34)	51.61 (35.24)	<0.001
ALT, U/L	39.05 (26.61)	38.43 (25.56)	41.33 (30.06)	<0.001
AST, U/L	63.81 (45.17)	62.27 (42.98)	69.49 (52.07)	<0.001
WBC, 10^9^/L	15.61 (9.48)	15.26 (8.86)	16.90 (11.38)	<0.001
Lymphocytes Count, 10^9^/L	1.07 (0.68)	1.12 (0.69)	0.90 (0.62)	<0.001
Comorbidity, No. (%)				
Hypertension	5041 (47.1)	4025 (47.8)	1016 (44.5)	0.006
Diabetes Mellitus	2817 (26.3)	2226 (26.4)	591 (25.9)	0.638
Cardiomyopathy	414 (3.9)	315 (3.7)	99 (4.3)	0.208
Acute Kidney Failure	4931 (46.0)	3456 (41.0)	1475 (64.6)	<0.001
Chronic Kidney Disease	2341 (21.9)	1752 (20.8)	589 (25.8)	<0.001
Chronic Pulmonary Disease	718 (6.7)	495 (5.9)	223 (9.8)	<0.001
Chronic Liver Disease	571 (5.3)	416 (4.9)	155 (6.8)	0.001
Vital Signs (mean ± SD)				
SBP, mmHg	114.94 (15.11)	115.56 (14.94)	112.66 (15.50)	<0.001
DBP, mmHg	61.66 (10.15)	61.83 (9.98)	61.02 (10.72)	0.001
MAP, mmHg	76.70 (10.04)	76.99 (9.85)	75.60 (10.66)	<0.001
Heart Rate, beat per min	87.56 (16.59)	86.53 (16.07)	91.33 (17.87)	<0.001
Resp Rate, breaths per min	20.10 (4.21)	19.69 (4.01)	21.59 (4.57)	<0.001
Temperature, (°C)	36.90 (0.62)	36.94 (0.56)	36.74 (0.79)	<0.001
Infection Site, No. (%)				
Bloodstream	1698 (15.9)	1338 (15.9)	360 (15.8)	0.932
Respiratory	1297 (12.1)	1008 (12.0)	289 (12.7)	0.381
Urinary	2004 (18.7)	1694 (20.1)	310 (13.6)	<0.001
Gastrointestinal	332 (3.1)	284 (3.4)	48 (2.1)	0.002
Peritoneal	93 (0.9)	82 (1.0)	11 (0.5)	0.034
Pleural	42 (0.4)	34 (0.4)	8 (0.4)	0.865
Skin And Soft Tissue	813 (7.6)	726 (8.6)	87 (3.8)	<0.001
HIV Infection, No. (%)	171 (1.6)	139 (1.6)	32 (1.4)	0.459
Chemotherapy, No. (%)	306 (2.9)	205 (2.4)	101 (4.4)	<0.001
Immunosuppressant Use, No. (%)	271 (2.5)	185 (2.2)	86 (3.8)	<0.001

SOFA, Sequential Organ Failure Assessment; BUN, blood uria nitrogen; ALT, alanine aminotransferase; AST, aspartate aminotransferase; INR, international normalized ratio; APTT, activated partial thromboplastin time; WBC, white blood cell; SBP, systolic blood pressure; DBP, diastolic blood pressure; MAP, mean arterial pressure; Resp Rate, respiratory rate; HIV, Human Immunodeficiency Virus.

Nonsurvivors exhibited significantly worse laboratory parameters except platelets compared to survivors (P < 0.001). Notable differences included lower ALC (0.90 ± 0.62×10^9^/L vs. 1.12 ± 0.69×10^9^/L, P < 0.001), higher bilirubin (3.08mg/dL vs. 1.96mg/dL, P < 0.001) and higher creatinine (2.13mg/dL vs. 1.74mg/dL, P < 0.001). Likewise, nonsurvivors were more likely to have hypertension, acute kidney failure, chronic kidney disease, chronic pulmonary disease, and chronic liver disease (P < 0.01 for all). With respect to vital signs, The mean systolic blood pressure (SBP) was significantly lower in nonsurvivors compared to survivors, as was the mean diastolic blood pressure. Additionally, nonsurvivors had higher heart rates and respiratory rates. Moreover, the analysis of infection sites showed similar distributions between survivors and nonsurvivors, with respiratory (12.1%), bloodstream (15.9%), and urinary infections (18.7%) being the most common. HIV infection rates showed no significant difference (1.6% vs 1.4%, P = 0.459). Chemotherapy (2.4% vs 4.4%, P < 0.001) and immunosuppressant use (2.2% vs 3.8%, P < 0.001) were significantly higher in nonsurvivors.

### 3.2 ALC is an independent prognostic predictor in sepsis patients

Both univariate and multivariate analyses were performed utilizing the Cox proportional-hazards model to examine the association between various clinical factors and the 28-day survival outcome in patients with sepsis (**Table 2**). Firstly, univariate Cox regression analysis was performed for each factor, revealing that several variables, including gender, age, SOFA score, laboratory data, comorbidities, vital signs, and infection sites were linked to an increased risk of death. Of particular interest, ALC emerged as a significant predictor. Patients exhibiting a lower ALC were found to be at a higher risk of mortality, with a Hazard Ratio (HR) of 0.62 (P < 0.001). Subsequently, variables with P value < 0.05 were incorporated into the multivariate analysis. After adjusting for other clinical variables, a lower ALC remained significantly associated with a higher risk of death (HR=0.88, P < 0.001). Further analysis confirmed that lower ALC is an independent predictor of mortality.

**Table 2:** Univariate and multivariate cox regression analyses for 28-day mortality of the patients with sepsis.

Characteristics	Univariate analysis			Multivariate analysis		
	HR	CI	p value	HR	CI	p value
Gender	0.91	0.83 - 0.98	0.019	1.01	0.89 - 1.14	0.905
Age	1.02	1.02 - 1.02	0.000	1.03	1.02 - 1.03	0.000
SOFA	1.17	1.15 - 1.18	0.000	1.10	1.08 - 1.12	0.000
Laboratory Data						
Lymphocytes Count	0.62	0.58 - 0.67	0.000	0.88	0.79 - 0.98	0.024
WBC	1.01	1.01 - 1.01	0.000	1.00	0.99 - 1.01	0.956
Hemoglobin	0.95	0.93 - 0.97	0.000	0.99	0.96 - 1.02	0.407
Platelets	1.00	1.00 - 1.00	0.576			
Albumin	0.68	0.63 - 0.73	0.000	0.83	0.75 - 0.91	0.000
Bilirubin	1.04	1.03 - 1.04	0.000	1.03	1.02 - 1.04	0.000
BUN	1.01	1.01 - 1.01	0.000	1.01	1.00 - 1.01	0.000
Creatinine	1.08	1.06 - 1.09	0.000	0.89	0.85 - 0.94	0.000
INR	1.14	1.12 - 1.16	0.000	1.04	1.00 - 1.08	0.026
PTT	1.01	1.01 - 1.01	0.000	1.00	1.00 - 1.00	0.019
ALT	1.00	1.00 - 1.00	0.000	0.99	0.99 - 1.00	0.000
AST	1.00	1.00 - 1.00	0.000	1.00	1.00 - 1.00	0.001
Vital Signs						
SBP	0.99	0.98 - 0.99	0.000	1.00	1.00 - 1.01	0.449
DBP	0.99	0.99 - 1.00	0.000	0.99	0.98 - 1.01	0.459
MAP	0.99	0.98 - 0.99	0.000	1.00	0.98 - 1.03	0.687
Heart Rate	1.02	1.01 - 1.02	0.000	1.01	1.01 - 1.02	0.000
Resp Rate	1.09	1.08 - 1.10	0.000	1.04	1.03 - 1.06	0.000
Temperature	0.62	0.58 - 0.65	0.000	0.65	0.59 - 0.72	0.000
Infection Site						
Bloodstream	0.99	0.88 - 1.10	0.794			
Respiratory	1.01	0.89 - 1.14	0.875			
Urinary	0.65	0.57 - 0.73	0.000	0.54	0.46 - 0.65	0.000
Gastrointestinal	0.63	0.47 - 0.84	0.002	0.63	0.42 - 0.93	0.020
Peritoneal	0.51	0.28 - 0.92	0.025	0.43	0.19 - 0.96	0.039
Pleural	0.84	0.42 - 1.68	0.622			
Skin And Soft Tissue	0.45	0.36 - 0.55	0.000	0.49	0.36 - 0.66	0.000
Comorbidity						
Diabetes Mellitus	0.97	0.89 - 1.07	0.558			
Hypertension	0.88	0.81 - 0.96	0.003	0.85	0.75 - 0.97	0.015
Acute Kidney Failure	2.35	2.16 - 2.56	0.000	1.15	1.00 - 1.32	0.047
Chronic Kidney Disease	1.28	1.16 - 1.40	0.000	0.98	0.84 - 1.16	0.901
Chronic Pulmonary Disease	1.61	1.40 - 1.85	0.000	1.37	1.13 - 1.65	0.001
Chronic Liver Disease	1.34	1.13 - 1.57	0.001	0.90	0.71 - 1.15	0.420
Cardiomyopathy	1.14	0.93 - 1.39	0.215			
HIV Infection	0.87	0.62-1.24	0.447			
Chemotherapy	1.68	1.38-2.05	0.000	1.24	0.89-1.71	0.200
Immunosuppressant Use	1.56	1.26-1.94	0.000	1.06	0.75-1.50	0.736

SOFA, Sequential Organ Failure Assessment; WBC, white blood cell; BUN, blood uria nitrogen; ALT, alanine aminotransferase; AST, aspartate aminotransferase; INR, international normalized ratio; APTT, activated partial thromboplastin time; SBP, systolic blood pressure; DBP, diastolic blood pressure; MAP, mean arterial pressure; Resp Rate, respiratory rate; HIV, Human Immunodeficiency Virus.

### 3.3 ALC-SOFA score development

**Figure 2:**
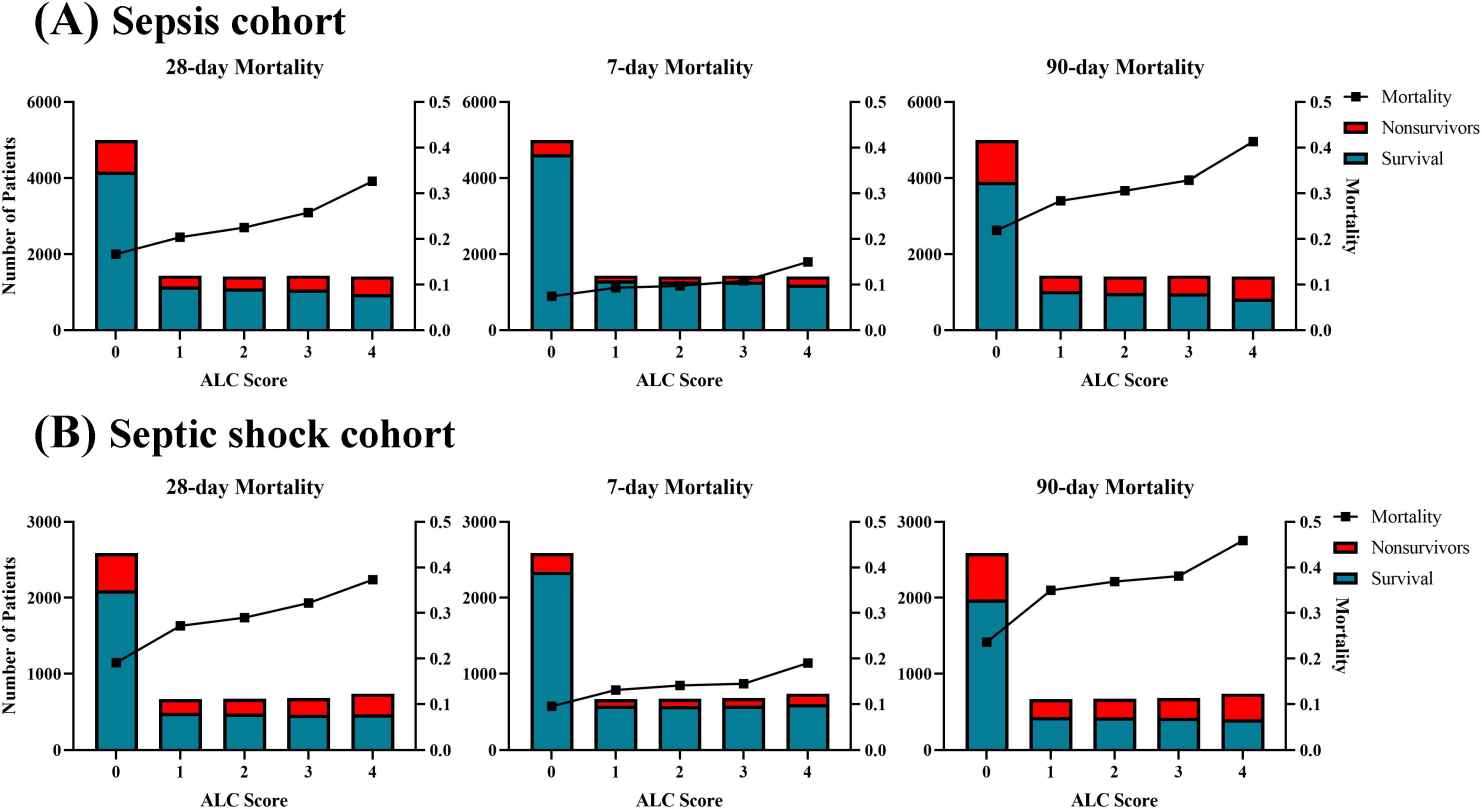
Distribution and corresponding mortality according to ALC score in the **(A)** sepsis cohort and **(B)** septic shock cohort. The mortality showed a linear increase with the ALC score.

The lymphocyte count (ALC) score was derived from lymphocyte count values. Usually, lymphopenia induced by sepsis is defined as a lymphocyte count below 1,000 cells/µL (Drewry et al., 2014; Podd et al., 2023; Wang et al., 2024). Therefore, a lymphocyte count greater than 1.0×10^9^/L was assigned a score of 0. The remaining values were divided into four categories according to the quartile of ALC: 1 (0.78×10^9^/L ≤ lymphocyte count < 1.0×10^9^/L), 2 (0.58×10^9^/L ≤ lymphocyte count < 0.78×10^9^/L), 3 (0.36×10^9^/L ≤ lymphocyte count < 0.58×10^9^/L) and 4 (lymphocyte count < 0.36×10^9^/L). The distribution of the ALC score and its association with 7-day, 28-day, and 90-day mortality rates in sepsis and septic shock patients are presented in **Figure 2**. Generally, the mortality rate increased with higher ALC scores in sepsis patients, suggesting that lower ALC are associated with poorer outcomes. Patients with a ALC score of 0 consistently exhibited the lowest mortality rate, while those with a score of 4 had the highest. For instance, the 28-day mortality rate ranged from 16.7% for a score of 0 to 32.7% for a score of 4 in sepsis patients, with similar trends observed for the 7-day and 90-day mortality rate. A similar pattern was also observed in septic shock patients.

**Figure 3:**
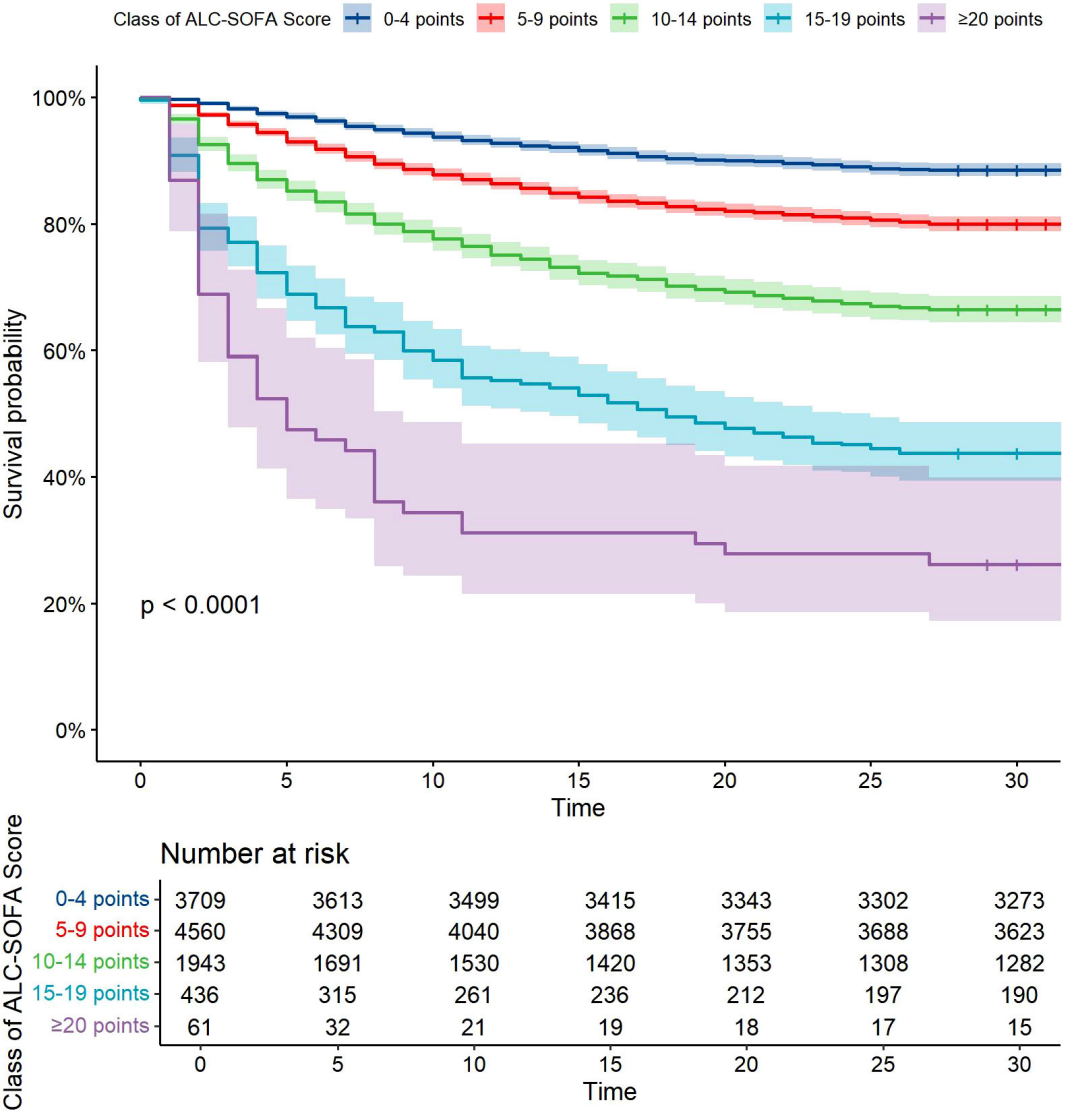
Kaplan-Meier survival curves with cumulative 28-day survival based on Class of ALC-SOFA score (by 5-point intervals). Categories differed significantly (P<0.0001, Log rank test).

Following this, we developed a modified SOFA score, referred to as the ALC-SOFA score, by combining the ALC score with the original SOFA score. The ALC-SOFA score (range: 0–28) was defined as the sum of the original SOFA score (range: 0–24) and the ALC score (**Table 3**). Kaplan-Meier survival curves stratified by different ALC-SOFA classes demonstrated that higher ALC-SOFA scores were associated with increased mortality (**Figure 3**).

**Table 3:** Sequential Organ Failure Assessment (SOFA) score with the absolute lymphocyte count (ALC) score.

Variable	Score				
	0	1	2	3	4
Respiratory system					
PaO_2_/FiO_2_	≥ 400	< 400	< 300	< 200 with respiratory support	< 100 with respiratory support
Hepatic system					
Bilirubin (mg/dL)	< 1.2	1.2–1.9	2.0–5.9	6.0–11.9	> 12.0
Cardiovascular system	MAP ≥ 70 mmHg	MAP < 70 mmHg	Dop < 5 or dob (any dose)^a^	Dop 5.1–15 or epi ≤ 0.1 or norepi ≤ 0.1^a^	Dop > 15 or epi > 0.1 or norepi > 0.1^a^
Coagulation					
Platelets × 10^3^/µL	≥ 150	< 150	< 100	< 50	< 20
Central nervous system					
Glasgow coma scale	15	13–14	10–12	6–9	< 6
Renal system					
Creatinine (mg/dL)	< 1.2	1.2–1.9	2.0–3.4	3.5–4.9	> 5.0
Urine output (mL/d)				< 500	< 200
Immune status					
(ALC score)					
lymphocytes count (10^9^/L)	>1	≥0.78and < 1	≥ 0.58 and < 0.78	≥0.36 and < 0.58	< 0.36

^a^Adrenergic agents administered for at least 1 h, all catecholamine doses represent μg/kg/min.

### 3.4 Comparison of predictive performance between ALC-SOFA and original SOFA score

**Figure 4:**
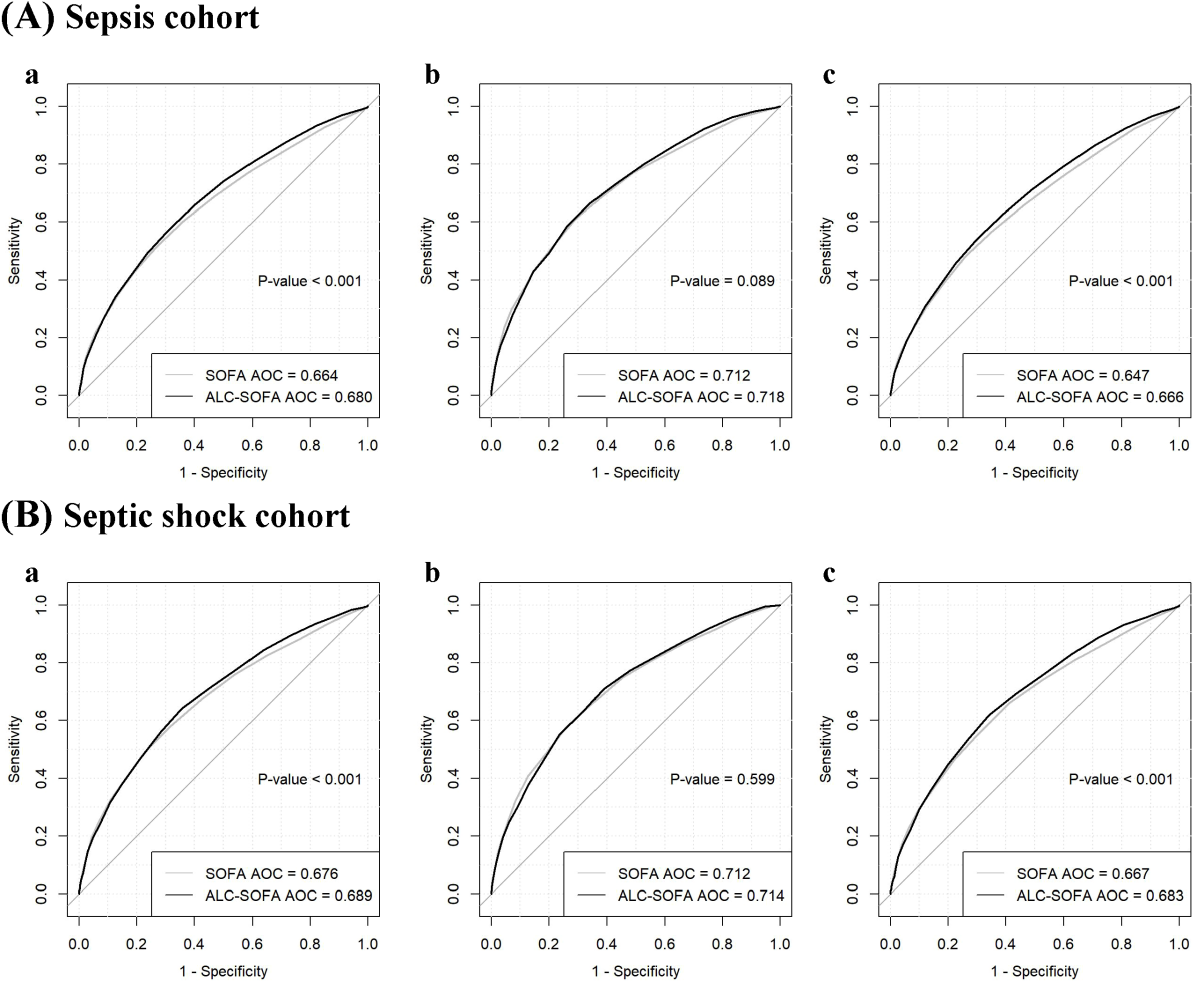
Receiver operating characteristic curves comparing the ability of ALC-SOFA and SOFA score to predict (a) 28-day mortality, (b) 7-day mortality and (c) 90-day mortality in the **(A)** sepsis cohort and **(B)** septic shock cohort.

In order to assess the predictive performance of the ALC-SOFA score and the original SOFA score for prognosis, we compared their respective areas under the receiver operating characteristic (AUROC) curves (**Figure 4**). The results indicated that the ALC-SOFA score showed a significant improvement in predictive accuracy for 28-day mortality compared to the original SOFA score (AUROC = 0.680 vs. 0.664, P < 0.001), as well as for 90-day mortality (AUROC = 0.666 vs. 0.647, P < 0.001). However, no significant difference was observed in the AUROC for 7-day mortality between the ALC-SOFA and original SOFA score (AUROC = 0.718 vs. 0.712, P = 0.089). Further analysis in the septic shock subgroup revealed similar findings. The ALC-SOFA score showed a greater ability to predict prognosis than the original SOFA score for 28-day and 90-day mortality, while the two scores demonstrated similar predictive performance for 7-day mortality (AUROC = 0.714 vs. 0.712, P = 0.599). Besides, we found that in the elderly patients subgroup (aged over 65), ALC-SOFA significantly outperformed SOFA in predicting 28-day, 7-day, and 90-day mortality. However, no significant improvement was observed in the immunocompromised individuals subgroup (including patients receiving chemotherapy, immunosuppressants, or with HIV infection) or in those with hematological disorders (such as leukemia, lymphoma, aplastic anemia, or myelodysplastic syndromes) (**Supplementary Figure S1**).

For 28-day mortality, the addition of ALC to SOFA yielded an NRI of 12.0% (P < 0.001) and an IDI of 0.5% (P = 0.022). For 7-day mortality, the NRI and IDI were 7.5% (P < 0.001) and -0.4% (P = 0.124). For 90-day mortality, the NRI and IDI were 13.1% (P < 0.001) and 0.8% (P < 0.001), respectively (**Table 4**). Additionally, we evaluated the performance of both the ALC-SOFA and original SOFA scores in predicting mortality at 28-day, 7-day, and 90-day intervals in patients suffering from sepsis and septic shock (**Table 5**). For sepsis patients, the ALC-SOFA score demonstrated higher sensitivity compared to the original SOFA score for predicting 28-day mortality (0.575 vs. 0.516), 7-day mortality (0.662 vs. 0.516), and 90-day mortality (0.543 vs. 0.482). Similarly, in septic shock patients, the ALC-SOFA score exhibited higher sensitivity than the original SOFA score for 28-day mortality (0.641 vs. 0.496), 7-day mortality (0.709 vs. 0.577), and 90-day mortality (0.618 vs. 0.560).

**Table 4:** AUC, NRI and IDI for evaluating improvement to predict mortality with ALC-SOFA versus SOFA score.

Outcome	Variable	ALC-SOFA (95% CI)	SOFA (95% CI)	P-value
28-day mortality	AUC	0.680 (0.668-0.693)	0.664 (0.651-0.677)	<0.001
	NRI	0.120 (0.084-0.148)		<0.001
	IDI	0.005 (0.001-0.009)		0.022
7-day mortality	AUC	0.718 (0.702-0.735)	0.712 (0.695-0.730)	0.089
	NRI	0.075 (0.031-0.111)		<0.001
	IDI	-0.004 (-0.009-0.001)		0.124
90-day mortality	AUC	0.666 (0.655-0.678)	0.647 (0.635-0.658)	<0.001
	NRI	0.131 (0.097-0.155)		<0.001
	IDI	0.008 (0.005-0.013)		<0.001

**Table 5:** Measures of performance of ALC-SOFA and original SOFA score for predicting mortality in patients with sepsis and septic shock.

Outcome	Variable	Sensitivity	Specificity	PPV	NPV	PLR	NLR
Sepsis 28-day Mortality	ALC-SOFA	0.575	0.687	0.332	0.857	1.839	0.618
	SOFA	0.516	0.731	0.342	0.848	1.916	0.663
7-day Mortality	ALC-SOFA	0.662	0.662	0.170	0.949	1.958	0.511
	SOFA	0.610	0.708	0.179	0.946	2.091	0.551
90-day Mortality	ALC-SOFA	0.543	0.699	0.411	0.797	1.801	0.655
	SOFA	0.482	0.740	0.419	0.786	1.856	0.700
Septic shock 28-day Mortality	ALC-SOFA	0.641	0.644	0.381	0.839	1.798	0.558
	SOFA	0.496	0.768	0.423	0.816	2.136	0.656
7-day Mortality	ALC-SOFA	0.709	0.611	0.206	0.936	1.823	0.476
	SOFA	0.577	0.740	0.240	0.925	2.219	0.571
90-day Mortality	ALC-SOFA	0.618	0.658	0.456	0.788	1.809	0.580
	SOFA	0.560	0.690	0.455	0.772	1.805	0.638

## 4 Discussion

The SOFA score was developed by the Working Group of the European Society of Intensive Care Medicine in 1996 and incorporated as one of the diagnostic criteria for sepsis-3 since 2016 (Vincent et al., 1996; Singer et al., 2016). Although the SOFA score is extensively used in clinical practice, certain aspects may have limitations as both clinical practice and the understanding of sepsis continue to evolve. Recent studies suggest that sepsis involves three immune classification strata: macrophage activation-like syndrome (MALS), intermediate, and immunoparalysis (Leventogiannis et al., 2022). The development of immunoparalysis, which is associated with an increased microbiological burden and death rate (Otto et al., 2011), is currently believed to be driven by multiple mechanisms, among which aberrantly induced cell death of immune cells is a major contributor (Boomer et al., 2011; Lamkanfi et al., 2009). Several studies have made significant efforts to identify the immunoparalysis phenotype in sepsis. For example, *Gómez* et al. proposed that immunoparalysis is classified by <5,000 HLA-DR receptors per CD14-monocyte (Leventogiannis et al., 2022). Researches have also investigated the utility of cytokines, mHLA-DR expression, and ALC in evaluating immunoparalysis (Samuelsen et al., 2024).

*Moreno* et al. proposed that several additional elements might be taken into consideration to develop a SOFA 2.0 (Moreno et al., 2023). We have noticed that, despite its utility, the current SOFA score does not take immune function into account, which is critical to understanding the pathophysiology of sepsis. As an immune function marker, lymphocytes play an important role in the immune response during sepsis (Wang et al., 2024). Patients with persistent low ALC are likely to be in an immunosuppressive state (Drewry et al., 2014; Pène et al., 2016; Stortz et al., 2018). According to the principles of SOFA score, the assessment of organ dysfunction or failure should be grounded on a concise set of simple, yet objective, variables that are readily and consistently measurable in every healthcare institution (Vincent et al., 1996). ALC satisfies this principle, as it is a clinically accessible and easily obtainable measure. Consequently, we sought to modify the SOFA score by incorporating ALC.

In this study, we developed a modified SOFA score, referred to as the ALC-SOFA score. The ALC-SOFA score, which exhibited higher AUROC and sensitivity, demonstrated superior predictive accuracy for 28-day and 90-day mortality compared to the original SOFA score. An observational retrospective study conducted by Liu et al. suggested that ALC on both day 5 and day 7 emerged as significant predictors of the 28-day mortality in patients with sepsis resulting from intra-abdominal infection (Liu et al., 2021). Another Retrospective cohort study indicated that dynamic changes in ALC are closely linked to 90-day mortality in sepsis patients (Chen et al., 2024). These previous studies confirmed our conclusions. Unlike previous modifications of the SOFA score, this study uniquely incorporates immune function, offering a biologically enriched scoring system. Additionally, we demonstrated that ALC is an independent prognostic predictor of sepsis outcomes. Our findings align with the previous studies, reinforcing the prognostic significance of ALC in sepsis (Wang et al., 2024). These findings highlight the potential of integrating immune biomarkers into clinical scoring systems to improve prognosis prediction and risk stratification for sepsis patients.

Interestingly, while the ALC-SOFA score outperformed the original SOFA score for predicting 28-day and 90-day mortality, it did not show a significant advantage for 7-day mortality. Previous study indicated that the death distribution of sepsis patients displayed a trimodal distribution, whose third upswing occurs approximately 60 to 90 days after sepsis and continues to soar as time progresses (Delano and Ward, 2016). Ongoing advancements in clinical treatment strategies have led to a higher survival among patients during the initial phase of inflammation and the accompanying anti-inflammatory response. However, many patients continue to exhibit different degrees of prolonged immune dysfunction and immune suppression that may be associated with long-term mortality. A study in community-acquired pneumonia (CAP) patients demonstrated that many patients are discharged with lingering subclinical inflammation even after clinical recovery, which is linked to a higher risk of mortality (Yende et al., 2008). Another observational study also suggested that patients with persistently aberrant markers of impaired host immunity have a greater incidence of chronic critical illness (Stortz et al., 2018). Similarly, *Inoue* et al. found that lymphopenia persisted for at least 21 days in elderly sepsis patients who did not survive (Inoue et al., 2013). Early immune suppression may contribute to later mortality but does not immediately impact short-term survival, as sepsis-induced immune dysfunction evolves. Furthermore, another potential explanation for our finding could be related to the duration of decreased ALC. In this study, we extracted the first-day ALC rather than the dynamic change of counts. The statistics showed that 74% of the population experienced lymphopenia on days 1-2, and this condition persisted in 56% of patients on days 6-8 (Zorio et al., 2017). Another study suggested that the initial ALC on day 1 was comparable between survivors and nonsurvivors in septic shock patients. However, the change in ALC from day 1 to day 6 demonstrated a significant disparity in the capacity to recover from the initial lymphopenia (Le Tulzo et al., 2002). *Heffernan* et al. found that patients who either failed to develop lymphopenia or who failed to appropriately resolve lymphopenia were associated with significantly higher mortality and shorter time to death (Heffernan et al., 2012). The ability to recover a normal ALC may be associated with 7-day mortality, but this aspect was not explored in this study. More detailed time frames, such as 14-day or 21-day mortality, may provide further insights into the role of ALC in sepsis prognosis. Additionally, sepsis is a complex and multifactorial disease. While immune dysfunction is a key aspect, other factors such as age, organ failure, hemodynamic instability, and medical intervention may play more significant roles in determining short-term survival (Tong et al., 2025).

Although ALC-SOFA showed a statistically significant advantage over SOFA in predicting 28-day and 90-day mortality, we noted that the absolute AUC improvement for 28-day mortality was modest (0.016). Given the substantial sample size, statistical significance alone may not be sufficient to support claims of meaningful improvement. Therefore, we further introduced NRI and IDI, which demonstrated that ALC-SOFA outperformed the original SOFA in predicting both 28-day and 90-day mortality. These findings indicate that the improved performance of ALC-SOFA reflects a true enhancement in predictive ability, rather than a statistically significant difference resulting solely from the large sample size. In addition, we noted that the improvement of ALC-SOFA over SOFA was not substantial. This modest improvement may be due to the fact that components of the ALC-SOFA score other than ALC also influence prognosis. The ease of availability and low cost of ALC support its potential value for incorporation into the SOFA score to enhance clinical prognostic performance.

Moreover, we also noted that the applicability of ALC-SOFA to specific subgroups needs to be evaluated. Our completed subgroup analyses demonstrated that ALC-SOFA significantly outperformed SOFA in predicting 28-day, 7-day, and 90-day mortality among patients over 65 years. However, this predictive improvement was not observed in immunocompromised individuals or patients with hematological disorders. This suggests that ALC-SOFA may not be applicable to immunocompromised individuals and patients with hematological disorders. One possible explanation is that immunosuppression and hematological disorders obscure the ALC changes caused by sepsis, making the ALC variation primarily reflect the progression of these underlying conditions rather than the immune status related to sepsis. This highlights the need for further investigation into the applicability of ALC-SOFA in specific subgroups, particularly when lymphopenia is caused by non-infectious factors.

Several limitations inevitably exist. Firstly, the data from the MIMIC-IV database were collected from one medical center, making this a retrospective, single-center study with unavoidable selection bias. Secondly, the development of the ALC score was based on the quartile of ALC, which may not represent the optimal grading criteria. Thirdly, while lymphocyte count serves as a valuable immune biomarker, the lack of inclusion of other markers, such as neutrophil-to-lymphocyte count ratio or immune cell subset, may limit the comprehensiveness of the scoring system. Fourthly, we only measured the first-day ALC-SOFA score, without assessing its dynamic changes during ICU stay and their association with outcomes. Finally, external validation in independent cohorts is needed to confirm the generalizability of ALC-SOFA stratification beyond our study population.

Future studies could validate the ALC-SOFA score in multicenter cohorts to assess its viability across different healthcare systems and explore the inclusion of other immune-related biomarkers to further refine its predictive power. Additionally, the role of lymphocyte subpopulations in immune suppression and association with clinical outcomes for sepsis patients could also be explored in the future, which could deepen our understanding of sepsis pathophysiology. Although this study used only the first-day ALC-SOFA score, dynamic changes in the ALC-SOFA score may provide stronger prognostic value. Further studies should explore optimal time points and intervals for lymphocyte monitoring and assessment of ALC-SOFA score.

In summary, the ALC-SOFA score incorporating ALC as an immune function marker provides a novel tool for predicting prognosis in sepsis patients and enhances the potential of immune markers in clinical applications.

## Data Availability

The raw data supporting the conclusions of this article will be made available by the authors, without undue reservation.
