# Supplementary material for: Enhancing prognostic accuracy in sepsis: a modified SOFA score incorporating lymphocyte count as an immune function marker
Source: Front Cell Infect Microbiol. 2025 Jul 31;15:1593589. doi: 10.3389/fcimb.2025.1593589 (PMC12350375; doi:10.3389/fcimb.2025.1593589)
Supplement: Supplementary file 1 [file Table1.docx]

Supplementary Material

# Supplementary Figures


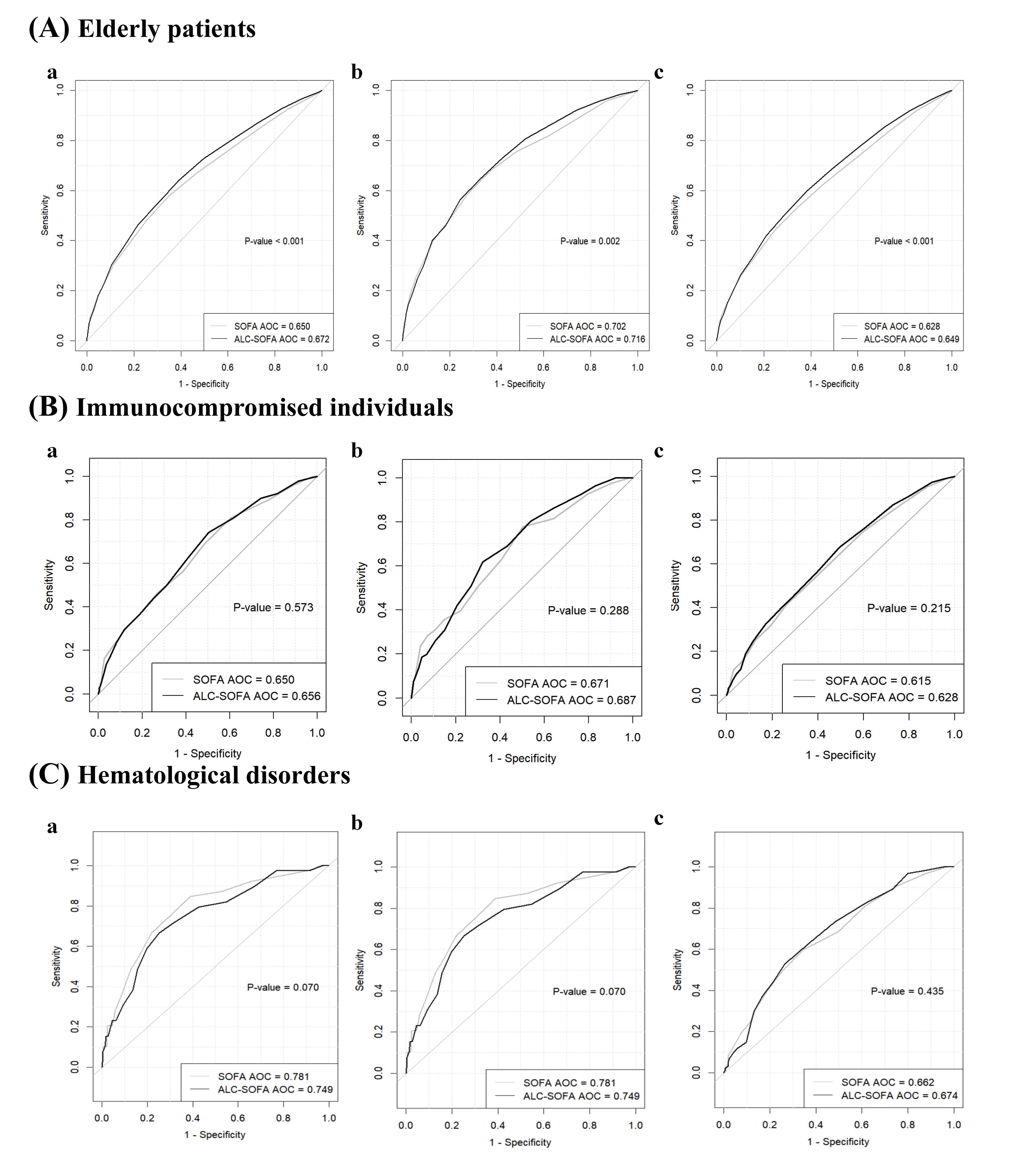


**Supplementary Figure 1.** Receiver operating characteristic curves comparing the ability of ALC-SOFA and SOFA score to predict (a) 28-day mortality, (b) 7-day mortality and (c) 90-day mortality in each cohort.
